# Maternal Infection Impairs Motor Coordination in an Experimental Meningitis Rat Model Through Altered MMP-2/3/9 Activity, H3K4 Trimethylation, and *Reln* Methylation

**DOI:** 10.3390/ijms27093761

**Published:** 2026-04-23

**Authors:** Tharmiya Sekar Surya, Swamynathan Sowndharya, Bhagavathi Sundaram Sivamaruthi, Chaiyavat Chaiyasut, Koilmani Emmanuvel Rajan

**Affiliations:** 1 Behavioural Neuroscience Laboratory, Department of Animal Science, Bharathidasan University, Tiruchirappalli 620024, India; surya.tss0206@gmail.com (T.S.S.); sowndharyaswamynathan.98@gmail.com (S.S.); 2 Innovation Center for Holistic Health, Nutraceuticals, and Cosmeceuticals, Faculty of Pharmacy, Chiang Mai University, Chiang Mai 50200, Thailand; sivamaruthi.b@cmu.ac.th; 3 Office of Research Administration, Chiang Mai University, Chiang Mai 50200, Thailand

**Keywords:** maternal infection, matrix metalloproteinases, epigenetic changes, reelin, motor coordination

## Abstract

Maternal infection (MI) can increase the risk of neurodevelopmental and behavioural changes. This study examined MI-induced changes in motor coordination through the inflammatory-pathway-mediated epigenetic status of *Reln*. On gestational day (GD) 10, rats were assigned as (i) Control (Ctrl); (ii) *Cronobacter sakazakii* (CS) infection on GD-10 through recto-vaginal colonization; (iii) Negative Control (NC) [infected with *C. sakazakii* and treated with dimethyl sulfoxide (DMSO) 1 h before and 24 h after infection]; and (iv) *C. sakazakii*-infected rats treated with matrix metalloproteinase inhibitor (MMPI), 1 h before and 24 h after infection (CS + MMPI). Offspring were subjected to footprint analysis and the ladder rung walking test, which revealed that MI caused significant deficits in motor coordination. In addition, MI activated complement components—a disintegrin and metalloproteinase with thrombospondin motifs-1 (ADAMTS-1, C5a)—as well as proinflammatory cytokines such as interleukin-6 (IL-6) and matrix metalloproteinases (MMP-2, MMP-3, and MMP-9). Furthermore, the levels of DNA methyltransferase 3 alpha (DNMT3A), methyl-CpG-binding protein 2 (MeCP2), and histone H3 lysine 4 trimethylation (H3K4me3) were elevated in the CS and NC groups. Concurrently, the level of *Reln* promoter methylation increased; as a result, mRNA and protein, as well as postsynaptic density protein-95 (PSD-95), levels were decreased. Overall, the findings suggest that MI altered MMP-2/3/9 activity, H3K4me3, and the methylation of *Reln*, thereby affecting reelin, synaptic protein expression, and motor coordination in an experimental meningitis rat model.

## 1. Introduction

Bacterial infections during pregnancy can cause neonatal meningitis, increasing the risk of neurodevelopmental and behavioural changes in the offspring [1,2,3]. Excessive maternal immune responses produced during maternal infection (MI) activate the complement system, releasing immune-inflammatory anaphylatoxins such as a disintegrin and metalloproteinase with thrombospondin motifs (ADAMTS, C5a) [4,5]. During pregnancy, elevated levels of ADAMTS-1, a proinflammatory peptide, can influence fetal development through the maternal–fetal interface [6,7]. As part of the immune response, proinflammatory cytokines initiate a signalling cascade that activates the nuclear factor kappa-light-chain-enhancer of activated B cells (NF-κB) [8]. Further, it undergoes nuclear translocation and promotes the expression of interferons (IFN-α, IFN-β), interleukins 6 (IL-6), tumour necrosis factor (TNF-α) [9,10,11], and matrix metalloproteinase (MMP-2, MMP-3, and MMP-9) [12,13]. Elevated matrix metalloproteinases (MMPs) facilitate the process of extracellular matrix (ECM) protein cleavage, including reelin, a large extracellular glycoprotein involved in synaptic plasticity [14], in which activated C5a is a core component [15,16]. Altered reelin levels during brain development disrupt neuronal communication and behaviour by affecting neuronal development and synaptic plasticity [17,18].

In addition, recent studies have highlighted that MI significantly alters the neural epigenetic landscape, neuronal migration, differentiation, and maturation in the developing brain [19,20]. DNA methyltransferases (DNMTs) and methyl CpG-binding protein-2 (MeCP2) are central to these processes. DNMTs catalyze the de novo methylation of CpG dinucleotides and repress gene expression [21,22], affecting neuronal development [23]. MeCP2 acts as a transcriptional repressor in a DNA methylation-dependent manner; either DNA demethylation or the removal of MeCP2 from its binding site suppresses repression [24,25]. Furthermore, MeCP2, as an epigenetic regulator, suppresses gene expression via interactions with di- and tri-methylated histone regions [26].

*Cronobacter sakazakii* (Enterobacteriaceae) is an opportunistic pathogen reported to cause life-threatening septicaemia and meningitis [27] and long-term neurological sequelae, including hearing, visual, motor defects, seizure and memory impairments, in neonates [28,29,30]. Considering these reports, the present study was designed. Previously, we demonstrated that *C. sakazakii* infection suppresses serotonin transporter (SERT) expression via microRNA-16-mediated regulation [31]. Subsequently, we found that elevated C5a activates mitogen-activated protein kinase kinase 1 (MEKK1). The activated MEKK1 pathway appears to modulate SERT expression, potentially through inflammatory cascades such as NF-κB–IL-6–IFN-α1 or via the apoptosis signal-regulating kinase (ASK)-1–c-Jun N-terminal kinase (JNK)-1/3–protein kinase B gamma (AKT3) axis [32]. Elevated levels of proinflammatory cytokines also facilitate the proteolytic cleavage of reelin, and through splicing [ApoER2 (Δ)/Ex-19] alter its receptor, apolipoprotein E receptor 2 (ApoER2). Reduced reelin alters the phosphorylation of its adaptor protein Disabled-1 (Dab1), resulting in the differential expression of N-methyl-D-aspartate (NMDA) receptor subunits NR2A and NR2B. These changes in NMDA receptor composition influence the postsynaptic density protein-95 (PSD-95), brain-derived neurotrophic factor expression, disrupting synaptic signalling and neuronal plasticity [33]. The inflammation-induced proteolytic cleavage of reelin at its functional domains has been implicated in reduced synaptic plasticity [33]. Moreover, inflammation-associated epigenetic modifications may further affect upstream (top-down) regulatory pathways. Therefore, the present study is designed to investigate whether MI altered the methylation status of the *Reln* promoter, thereby altering its expression, as well as synaptic proteins and motor coordination, in a maternal infection-mediated experimental meningitis rat model.

## 2. Results

### 2.1. Maternal Infection Impairs Motor Coordination in Their Offspring

**Figure 1:**
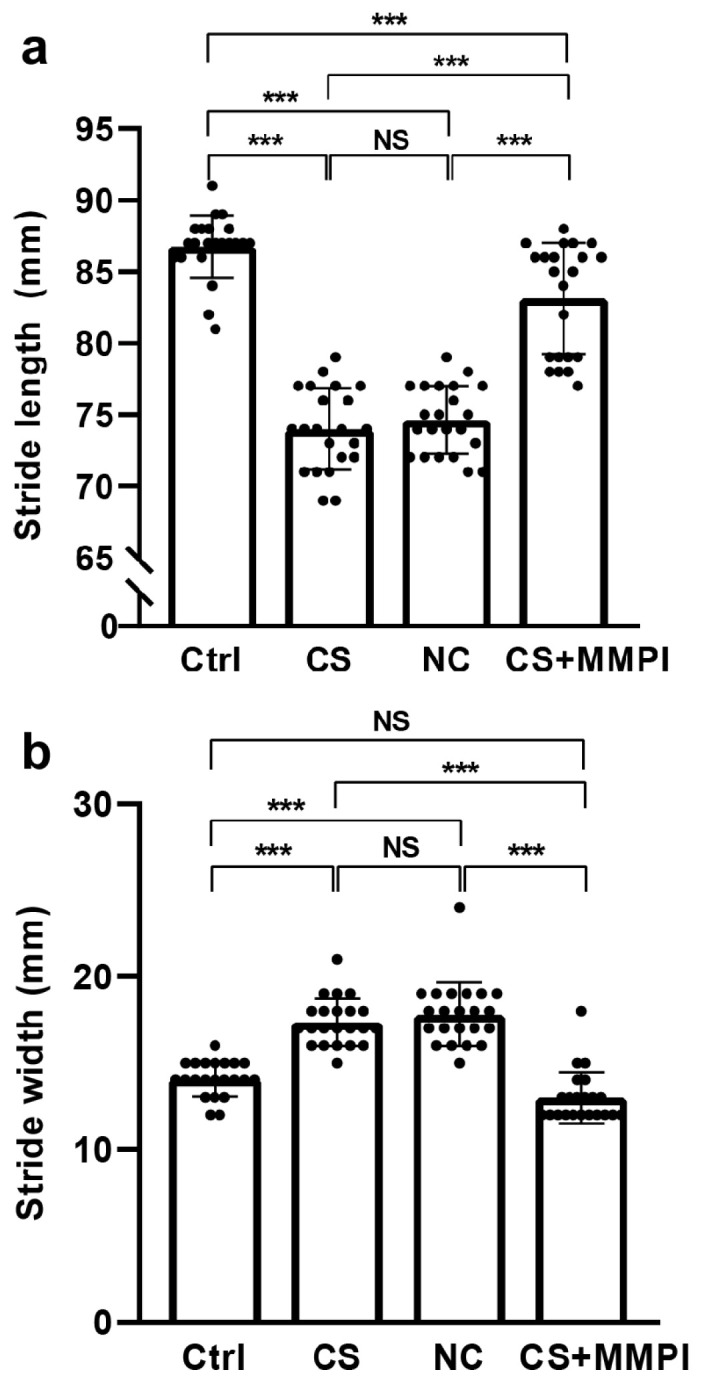
Footprint test analysis showing that maternal infection (MI) alters gait patterns in male adolescent offspring. (**a**) Stride length was significantly reduced in *C. sakazakii* (CS)-infected and Negative Control (NC) groups compared to Control (Ctrl) and CS + MMPI-treated groups. (**b**) Stride width was significantly increased in the CS and NC groups compared to the Ctrl and CS + MMPI-treated groups. Values represent mean ± SEM (Ctrl, *n* = 22; CS, *n* = 22; NC, *n* = 22; CS + MMPI, *n* = 22). Significant difference indicated as *** *p* < 0.001; non-significant differences are indicated as NS.

We have assessed gait pattern using footprint analysis, and significant differences in stride length (SL) were observed among groups (F_(3,87)_ = 104.13, *p* < 0.001). Post hoc analyses revealed that SL was significantly shorter in the CS and NC groups than in the Ctrl/CS + MMPI groups. Significant difference was not found between the CS and NC group, whereas SL was significantly shorter in the CS + MMPI group than the Ctrl group (Figure 1a; Supplementary Table S3).

Similarly, a significant difference was detected in the stride width (SW) among groups (F_(3,87)_ = 58.99, *p* < 0.001). SW was significantly wider in the CS NC groups than in the Ctrl and CS + MMPI groups (*p* < 0.001), but significant differences were not observed between the CS and NC or Ctrl and CS + MMPI groups (Figure 1b; Supplementary Table S3). These findings indicate that CS infection disrupts gait parameters, resulting in reduced SL and increased SW, and that MMPI administration partially ameliorates these MI-induced gait alterations.

**Figure 2:**
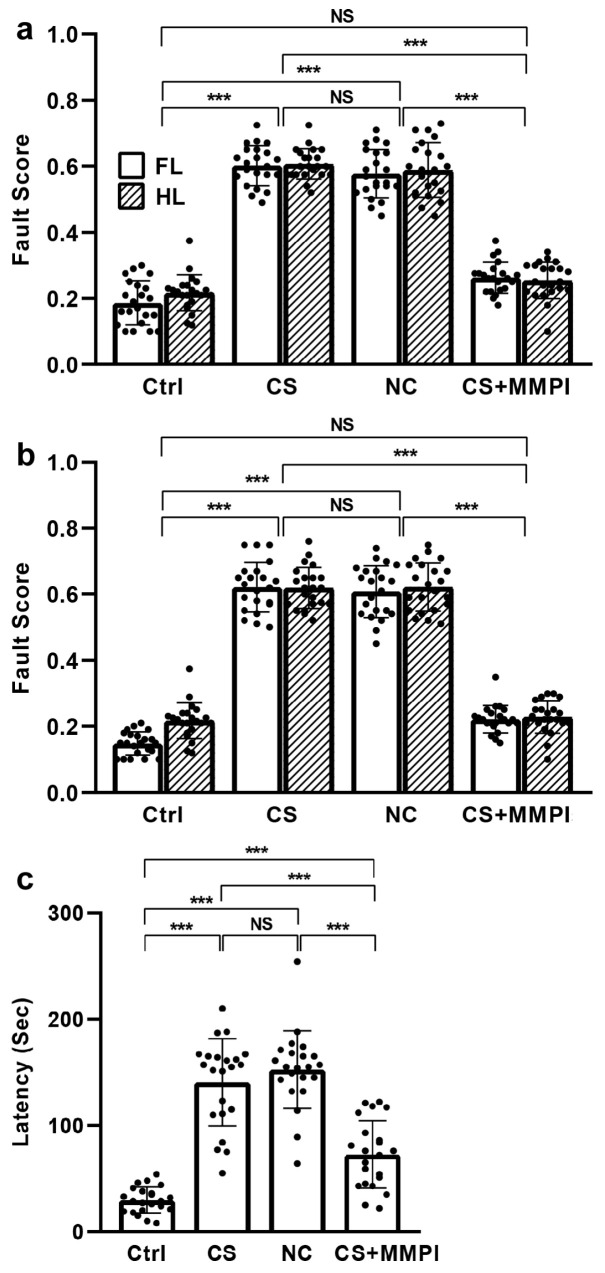
Ladder rung walking test showing that maternal infection (MI) impairs skilled limb coordination in male adolescent offspring. *C. sakazakii* (CS)-infected and Negative Control (NC) groups showed significantly more misplacements during (**a**) training, (**b**) testing and (**c**) latency compared to Control (Ctrl) and CS + MMPI-treated groups. Values represent mean ± SEM (Ctrl, *n* = 22; CS, *n* = 22; NC, *n* = 22; CS + MMPI, *n* = 22). Significant difference indicated as *** *p* < 0.001; NS indicates non-significant differences.

We evaluated limb coordination using the ladder rung walking test. During training, significant group differences were evident (F_(3,175)_ = 517.33, *p* < 0.001), but no differences were found between forelimb and hind limb performance (F_(1,175)_ = 1.10, *p* = 0.295) or in their interaction (F_(3,175)_ = 0.71, *p* = 0.542). Post hoc tests revealed higher fault scores (indicating more missteps) in the CS and NC groups than in the Ctrl and CS + MMPI groups, but no differences between the CS and NC groups or Ctrl and CS + MMPI groups (Figure 2a; Supplementary Table S4).

During testing, group differences remained significant (F_(3,175)_ = 680.27, *p* < 0.001), with modest differences between forelimb and hind limb performance (F_(1,175)_ = 5.75, *p* = 0.018) and a significant interaction (F_(3,175)_ = 3.04, *p* = 0.031). Post hoc analyses confirmed higher fault scores in the CS and NC groups than in the Ctrl and CS + MMPI groups, but no differences between the CS and NC or Ctrl and CS + MMPI groups (Figure 2b; Supplementary Table S4). Collectively, these results demonstrate MI-induced impairments in limb coordination, a finding which is validated by MMPI treatment. Further, the effect of body weight on locomotor behaviour was analyzed. The Pearson’s correlation analysis (Supplementary Figure S2) analyses revealed that the body weight did not influence the gait parameters, i.e., stride length (Ctrl: r = 0.15; CS: r = 0.12; NC: r = 0.13; CS + MMPI: r = 0.05) and stride width (Ctrl: r = 0.23; CS: r = 0.11; NC: r = 0.17; CS + MMPI: r = 0.04), and significant difference was not found between groups.

### 2.2. Maternal Infection Induces Expression of C5a and IL-6

**Figure 3:**
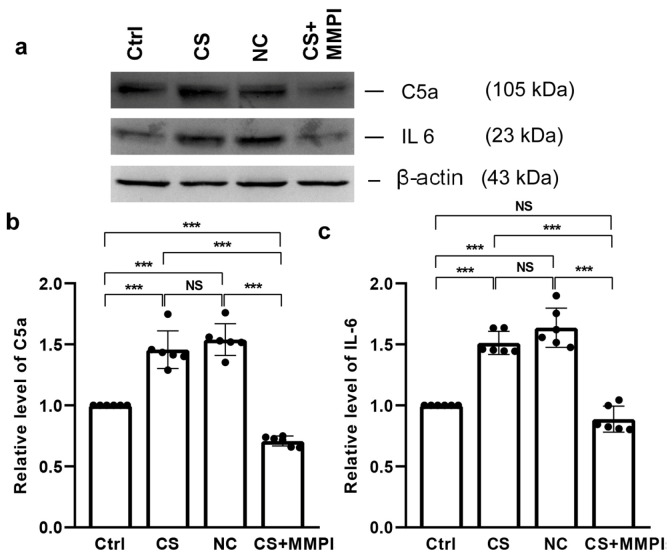
Maternal infection (MI) increases the expression of C5a and IL-6. (**a**) Representative Western blots showing immunoreactivity for anti-C5a and anti-IL-6. MI increased the expression of (**b**) C5a and (**c**) IL-6 in *C. sakazakii* (CS)-infected and Negative Control (NC) groups compared to Control (Ctrl) and CS + MMPI-treated groups. Values represent mean ± SEM (*n* = 6/group). Significant difference indicated as *** *p* < 0.001; NS indicates non-significant differences.

MI significantly altered C5a levels among groups (F_(3,23)_ = 87.21, *p* < 0.001). In comparison, the level of C5a was elevated in the CS and NC groups compared to the Ctrl and CS + MMPI groups. Notably, a reduced level was found in the CS + MMPI group compared to the Ctrl group, but a significant difference was not detected between the CS and NC groups (Figure 3a,b; Supplementary Table S4). Similarly, IL-6 levels significantly differed across groups (F_(3,23)_ = 71.46, *p* < 0.001), with higher levels in the CS and NC groups than in the Ctrl and CS + MMPI groups. But observed differences were not significant between CS and NC groups or Ctrl and CS + MMPI groups (Figure 3c; Supplementary Table S5, Supplementary Figure S3).

### 2.3. Maternal Infection Increases Expression of Matrix Metalloproteinases 2, 3 and 9

**Figure 4:**
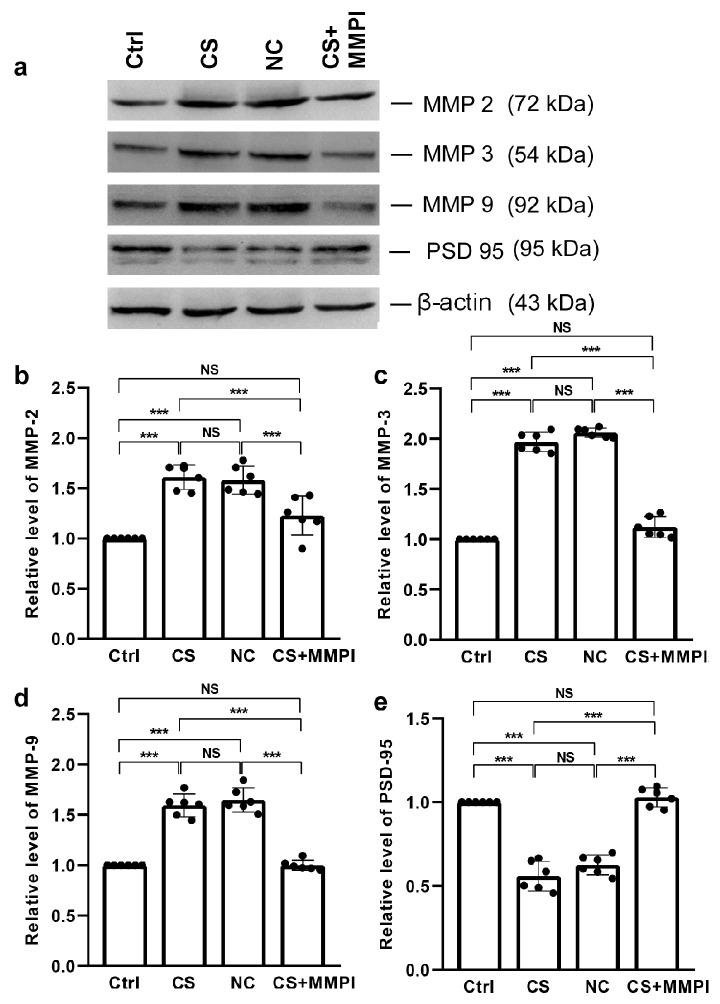
Maternal infection (MI) alters the expression of MMP-2, MMP-3, MMP-9, and PSD-95. (**a**) Representative Western blots showing immunoreactivity for anti-MMP-2, anti-MMP-3, anti-MMP-9, and anti-PSD-95. MI increased the expression of (**b**) MMP-2, (**c**) MMP-3, (**d**) MMP-9, and decreased (e) PSD-95 expression in *C. sakazakii* (CS)-infected and Negative Control (NC) groups compared to Control (Ctrl) and CS + MMPI-treated groups. Values represent mean ± SEM (*n* = 6/group). Significant difference indicated as *** *p* < 0.001; NS indicates non-significant differences.

Levels of MMP-2, MMP-3, and MMP-9 expression were significantly altered across groups [MMP-2: F_(3,23)_ = 34.90, *p* < 0.001; MMP-3: F_(3,23)_ = 332.24, *p* < 0.001; MMP-9: F_(3,23)_ = 104.44, *p* < 0.001]. The pattern of MI-induced upregulation was consistent across these MMPs, with significantly higher expression in CS and NC groups than Ctrl and CS + MMPI groups. No differences were found between the NC and CS or Ctrl and CS + MMPI groups (Figure 4a–d; Supplementary Table S6, Supplementary Figure S4).

### 2.4. Maternal Infection Reduces the Expression of Post-Synaptic Density (PSD)-95

MI significantly reduced PSD-95 expression (F_(3,23)_ = 100.302, *p* < 0.001) in the CS and NC groups compared to the Ctrl and CS + MMPI groups, with no differences between the CS and NC groups or Ctrl and CS + MMPI groups (Figure 4e; Supplementary Table S6, Supplementary Figure S4).

### 2.5. Maternal Infection Altered the MeCP2, DNMT3A, and H3K4me3 Levels

**Figure 5:**
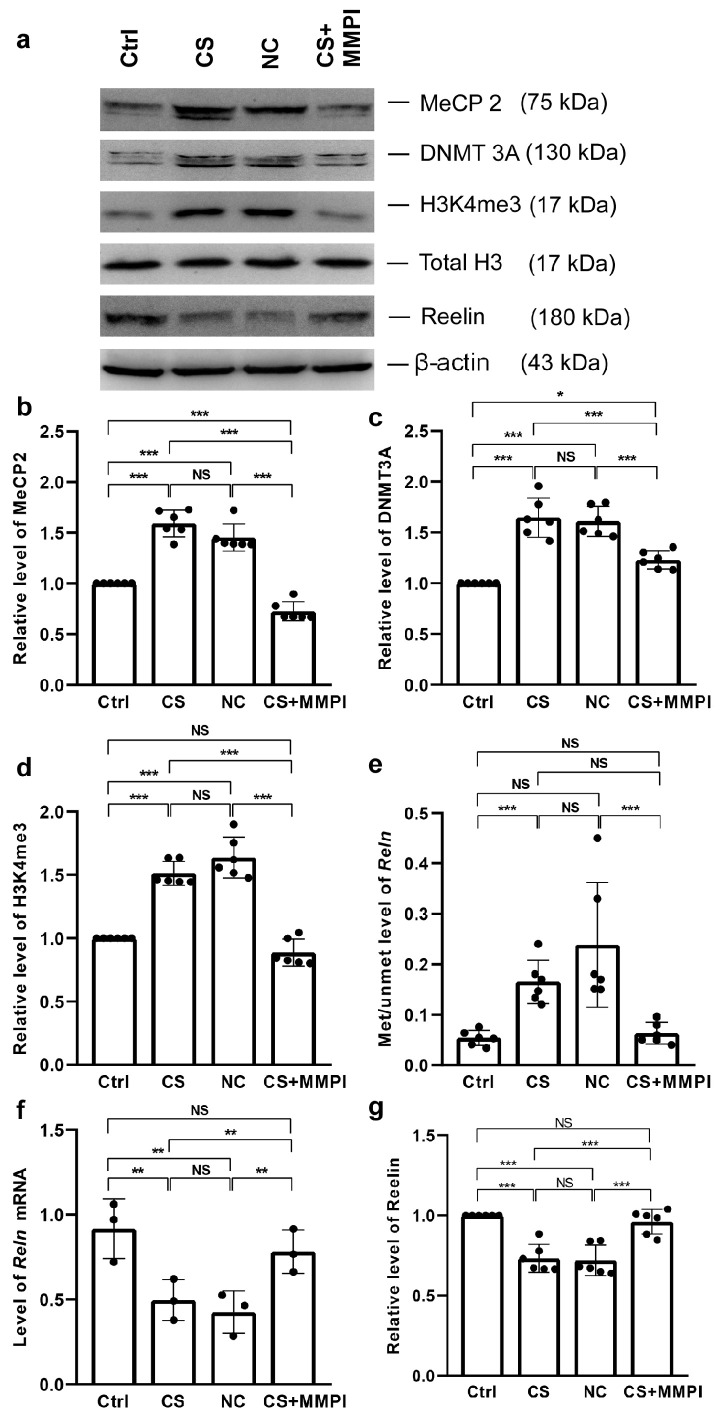
Maternal infection (MI) alters MeCP2, DNMT3A, and H3K4me3 expression, increases *Reln* promoter methylation, and reduces *Reln* mRNA and protein levels. (**a**) Representative Western blots depicting immunoreactivity for anti-MeCP2, anti-DNMT3A, anti-H3K4me3, total anti-H3, and anti-reelin. MI increased (**b**) MeCP2, (**c**) DNMT3A, (**d**) H3K4me3 expression, and (**e**) *Reln* promoter methylation, while decreasing (**f**) *Reln* mRNA and (**g**) reelin protein levels in *C. sakazakii* (CS)-infected and Negative Control (NC) groups compared to Control (Ctrl) and CS + MMPI-treated groups. Values represent mean ± SEM (*n* = 6/group). Significant difference indicated as * *p* < 0.05, ** *p* < 0.01, *** *p* < 0.001; NS indicates non-significant differences.

MI-induced alterations were evident in the methylation regulators MeCP2 and DNMT3A, with significant group differences [MeCP2: F_(3,23)_ = 88.05, *p* < 0.001; DNMT3A: F_(3,23)_ = 165.29, *p* < 0.001] (Figure 5a,b). Levels were elevated in the CS and NC groups compared to the Ctrl and CS + MMPI groups. But the difference between the CS and NC groups was not significant. However, the level of DNMT3A was higher in the CS + MMPI group than in the Ctrl group, whereas MeCP2 levels were lower (Figure 5c). H3K4me3 levels also differed significantly among groups (F_(3,23)_ = 247.67, *p* < 0.001), with elevations in the CS and NC groups relative to the Ctrl and CS + MMPI groups, but no difference was found between the CS and NC or Ctrl and CS + MMPI groups (Figure 5d, Supplementary Table S7, Supplementary Figure S5).

### 2.6. Maternal Infection Increases Methylation in the Reln Promoter and Decreases Reelin Expression

*Reln* promoter methylation differed significantly among groups (F_(3,23)_ = 30.76, *p* < 0.001), with higher methylation in the CS and NC groups than in the Ctrl and CS + MMPI groups and no differences between the Ctrl and CS + MMPI or CS and NC groups (Figure 5e, Supplementary Table S7). *Reln* mRNA expression also varied among groups (F_(3,11)_ = 8.36, *p* = 0.008); the level of *Reelin* mRNA was lower in the CS and NC groups than in the Ctrl and CS + MMPI groups, but no difference was observed between the Ctrl and CS + MMPI or CS and NC groups (Figure 5f). Consistent with this, the level of reelin protein significantly differed among groups (F_(3,23)_ = 23.23, *p* < 0.001), with reductions in the CS and NC groups relative to Ctrl and CS + MMPI groups. However, no differences were present between the Ctrl and CS + MMPI or the CS and NC groups (Figure 5g, Supplementary Figure S4).

## 3. Discussion

Earlier, we have demonstrated that maternal infection (MI) alters the neuron and spine morphology and induces a deficit in odour recognition memory and depressive-like motor behaviour in the offspring, while MMPI inhibitor treatment reverses the effect [34]. In this study, MI-induced changes in reelin (Reln) methylation, its downstream expression, and motor coordination in offspring were examined. Our footprint analysis revealed that MI significantly altered gait parameters, characterized by reduced stride length (SL) and increased stride width (SW) in the *C. sakazakii* (CS)-infected and normal control (NC) groups compared to controls and MMP inhibitor (MMPI)-treated animals. The reduction in SL indicates that MI likely impairs limb coordination, whereas the increased SW may represent a compensatory mechanism to enhance postural stability [35,36]. Consistently, the ladder rung walking test demonstrated higher fault scores in the CS and NC groups, suggesting impaired accuracy in fore- and hind-limb placement, interlimb coordination, and balance [37]. Earlier studies documented that damage or disorder associated with cerebellum alters the muscle tone, motor timing prediction, errors in limb movement position, directional tuning and their movement speed [38,39]. These studies suggest the possible correlation between footprint and ladder rung performance supports that MI-induced damage disrupts motor coordination, which was rescued by MMPI treatment. Further molecular analysis may provide additional insight into the molecular mechanism. On the other side, an animal’s body weight is known to influence locomotor behaviour, including gait pattern and motor coordination [40,41]. We did not detect any significant difference in body weight; therefore, the observed impairment in locomotor behaviour could be linked to the pathological condition of infection. It is important to note that this study was performed in animals younger than PND-30; however, long-term follow-up, along with the progressive gain of body weight and progression of the pathological condition, may differ from the present observation.

The activation of the complement component C5a plays a crucial role in the regulation of innate inflammatory responses [42]. Excessive C5a production during pregnancy is known to contribute to inflammatory pathologies, including sepsis [43,44]. Consistent with this, we observed elevated C5a levels in the CS and NC groups compared to controls and MMPI-treated animals. This elevation may reflect the maternal immune programming of offspring immunity via transplacental cytokine transfer, potentially enhancing immune preparedness against pathogens. However, the bidirectional transfer of inflammatory mediators across the placenta may either enhance immune reactivity or promote tolerance [45,46]. Moreover, the C5a-mediated induction of interleukin-6 (IL-6) was observed in the CS and NC groups, indicating an active inflammatory response [47]. Pro-inflammatory cytokine IL-6 has been widely implicated in mediating MIA-induced neurodevelopmental alterations [48].

MMPs are zinc-dependent endopeptidases involved in extracellular matrix remodelling and neuroinflammatory processes. Increased levels of MMP-2, MMP-3, and MMP-9 were detected in the CS and NC groups compared to control and MMPI-treated animals. Such increases may result from cytokine-mediated induction (e.g., via IL-6) or blood–brain barrier (BBB) disruption [49,50]. Various brain cell types can express and secrete MMPs [51], which are known to degrade BBB components [52], myelin basic protein (MBP) [53], and immunogenic MBP peptides [54]. Specifically, MMP-2 contributes to BBB breakdown during bacterial meningitis [55], MMP-3 disrupts neuron–glia interactions and increases susceptibility to neurodevelopmental disorders [56], and excessive MMP-9 activity destabilizes long-term potentiation and synaptic homeostasis [57]. Conversely, the pharmacological inhibition of MMPs has been shown to mitigate brain injury and restore neuronal function [58,59]. In agreement with these findings, our data showed the role of MMPs.

Excessive inflammatory signalling during pregnancy can also reprogram the epigenome, leading to altered neurodevelopmental trajectories in offspring [60,61]. Consistent with this, we observed elevated levels of DNMT3A, MeCP2, and H3K4me3 in the CS and NC groups, which may reflect increased S-adenosylmethionine (SAM)-dependent methylation activity [62]. The hypermethylation of the *Reln* promoter was accompanied by reduced *Reln* mRNA and protein expression in these groups. In line with our observation, the altered expression of reelin in genetically knocked-out reeler mice has been reported to disrupt the organization of neuronal layers in the cortex, hippocampus and cerebellum by affecting pyramidal layers and Purkinje cells [17,63]. Further studies state that the specific inhibition of cortex-dependent signalling impairs motor function [64]. Supporting this, earlier we have reported structural changes in the cortex Purkinje neuron axonal bending, dendritic branching and defects in the maturation of spine [65]. Reelin plays a vital role in neuronal migration, synaptic organization, and motor coordination; thus, its downregulation during early postnatal development can contribute to neurological and motor impairments [66]. Furthermore, reelin interacts with PSD-95 through ApoER2 signalling [67,68,69]. As expected, reduced PSD-95 levels were observed alongside diminished reelin expression, supporting a link between MI, impaired synaptic plasticity, and behavioural deficits [70], which could be linked with the MMP9 mutant animal displaying reduced synaptic plasticity and cognitive impairment [71]. In neurobehavioural studies, little evidence is documented that females exhibit more variations, suggesting a need to use both sexes for any behavioural analysis [72,73]. However, only adolescent male animals were analyzed, partly based on the concern that oestrous cycle-associated variations may occur in females. This is the limitation of our study.

## 4. Materials and Methods

### 4.1. Animal

Timed pregnant rats (*Rattus norvegicus*) were maintained at standard conditions (12 h light/dark cycle; temperature, 26 ± 2 °C; humidity, 50 ± 5%) with ad libitum access to standard chow pellets and water throughout the experiment. All experimental procedures were reviewed and approved (BDU/IAEC/P27/2024, dated 23 March 2024) by the Institutional Animal Ethics Committee following the guidelines of the Committee for the Control and Supervision of Experiments on Animals (CPCSEA), India.

### 4.2. Bacterial Strain

*C. sakazakii* (ATCC BAA-894) strain was kindly provided by Prof. K. Balamurugan (Alagappa University, Karaikudi, Tamil Nadu, India). The strain was cultured on HiCrome™ *Enterobacter sakazakii* agar (Cat. #M1577; HiMedia, Thane, India), and the presence of *C. sakazakii* was confirmed by the formation of blue-green colonies. A single colony was selected, inoculated into Luria–Bertani (LB) broth (Cat. #M1245; HiMedia) to obtain 10^7^ Colony Forming Units (CFU). *C. sakazakii* culture (20 μL) was suspended in phosphate-buffered saline (PBS) and administered intravaginally to induce maternal rectovaginal colonization [74].

### 4.3. Experimental Groups

Pregnant rats (*n* = 6/group) were assigned to (i) Control (Ctrl); (ii) *C. sakazakii* infection (CS) [received a single intravaginal dose of 10^7^ CFU on GD-10]; (iii) Negative Control (NC), which received 50 μL of 5% dimethyl sulfoxide (DMSO) intraperitoneally 1 h before infection on GD-10 and 24 h after infection on GD-11; (iv) *C. sakazakii* + Matrix Metalloproteinase Inhibitor (CS + MMPI), which received Batimastat (50 mg/kg, Cat. #CAS130370-60-4; Calbiochem, Sigma-Aldrich, St. Louis, MO, USA) dissolved in 5% DMSO intraperitoneally 1 h before infection on GD-10 and 24 h after intravaginal *C. sakazakii* administration on GD-11 [58] (Supplementary Figure S1). An earlier study reported that early gestation (GD-10) effectively induces immune activation and alters the behaviour of offspring [75]. Therefore, we infect the pregnant rats during GD-10. After parturition, litters were housed with their dams in standard laboratory cages. Pups were weaned on postnatal day (PND) 24, then housed (3–4 littermates/cage) in standard laboratory conditions.

### 4.4. Behavioural Tests

#### 4.4.1. Footprint Analysis

The apparatus consisted of a transparent plexiglass runway (90 × 10 × 7 cm) with the floor covered in grid paper. On PND 27, male adolescent rats’ forepaws and the hind paws of each experimental animal [Ctrl (*n* = 22), CS (*n* = 22), NC (*n* = 22), CS + MMPI (*n* = 22)] were coated with nontoxic colored dye. Animals were allowed to traverse the runway individually from one end to the other. Parameters such as stride length (SL) and stride width (SW) were examined [76].

#### 4.4.2. Horizontal Ladder Rung Walking Test

The rung walking apparatus was constructed as described previously [77] and elevated to a height of 30 cm. Experimental rats [Ctrl (*n* = 22), CS (*n* = 22), NC (*n* = 22), CS + MMPI (*n* = 22)] were trained individually during early adolescence (PND 27–29) to traverse the ladder, which featured a symmetric pattern of 1–40 rungs spaced 2 cm apart, from a neutral starting platform to the end. On PND 30, rats were tested on an irregular (asymmetric) rung pattern (rungs at positions 15, 17, 19, 21, 23, 25, 27, 30, 31, 34, and 35). Limb placements on the rungs were video-recorded and scored using a standard quantitative foot-fault scoring system [65,78].

### 4.5. Sample Collection

After the completion of behaviour, animals were euthanized. The whole brain (*n* = 6/group) was carefully removed and placed on a prechilled ice plate, and the cortex was dissected. Cortex samples were stored at −80 °C and aliquoted for genomic DNA, total RNA, and total and histone protein isolation.

### 4.6. DNA Methylation Assay

Genomic DNA (*n* = 6/group) was isolated using a commercial kit (Cat. #FATGEM-001B; Tissue Genomic DNA Extraction Kit, Favorgen, Ping Tung, Taiwan), quantified (BioPhotometer Plus; Eppendorf, Hamburg, Germany), and processed for bisulfite conversion (2 μg/sample) using a bisulfite modification kit (Cat. #59104, EpiTect^®^ Bisulfite Kit, Qiagen, Venlo, The Netherlands).

### 4.7. RNA Isolation and cDNA Synthesis

Total RNA (*n* = 6/group) was isolated using TRIzol reagent (Cat. #7326890; PureZOL^TM^ RNA Isolation Reagent; Bio-Rad Laboratories, Hercules, CA, USA) and quantified, and samples (RNA 1.0 μg) were reverse-transcribed into cDNA using a commercial kit (Cat. #170-8891; Bio-Rad Laboratories, Hercules, CA, USA).

### 4.8. Quantitative Real-Time PCR (qRT-PCR)

Quantitative real-time PCR (qRT-PCR) was performed using a CFX96 Touch Real-Time PCR Detection System (Bio-Rad Laboratories, Hercules, CA, USA) with SYBR^®^ Green (Cat. #1725125; Bio-Rad Laboratories, Hercules, CA, USA). The DNA methylation level of *Reln* promoter was performed using bisulfite-modified DNA (0.2 ng) with methylated/unmethylated specific primers (50 pM each) [Supplementary Tables S1, S3 and S5] using the following conditions: 95 °C, 3 min; 40 cycles 95 °C, 15 s, 64 °C (unmethylated) or 54.2 °C (methylated), 1 min, and 72 °C, 15 s.

*Reln* expression was determined by performing reactions containing cDNA (0.2 μg) and specific primers [(200 pM), *Reln*/β-actin (internal control)] [79]. Cycling conditions were as follows: 94 °C for 30 s; 40 cycles of 94 °C for 5 s, 66 °C (*Reln*) or 65 °C (*β-actin*) for 1 min, and 72 °C for 5 s. The specificity of the amplification was confirmed by melt-curve and dissociation-curve analysis. Each reaction was performed in triplicate and verified by 10% native polyacrylamide gel electrophoresis. Data were normalized to the internal control (*β-actin*) and expressed as mean fold change.

### 4.9. Total Protein Isolation

Cortical tissue (*n* = 6/group) was homogenized in ice-cold lysis buffer containing protease inhibitor. Lysates were incubated on ice (30 min) and centrifuged at 12,000× *g* for 30 min at 4 °C. Supernatant was collected and recentrifuged (12,000× *g*, 30 min) at 4 °C. Protein concentration was quantified (Cat. #5000006; Bio-Rad Protein Assay Kit) and stored at −80 °C [28].

### 4.10. Histone Protein Isolation

Cortical tissue (*n* = 6/group) was homogenized in TX buffer, incubated on ice (15 min) and centrifuged (2000× *g*, 10 min) at 4 °C. The supernatant was discarded, and then the pellet was resuspended in TX buffer with 0.2 M HCl. This suspension was incubated on ice (30 min) and centrifuged (10,000× *g*, 10 min) at 4 °C. The supernatant was quantified as mentioned above and stored at −80 °C as aliquoted.

### 4.11. Western Blot

Total protein (40 μg; *n* = 6/group) was resolved in polyacrylamide gel (8% or 10%), then transferred onto PVDF membranes (Cat. #1620177; Immuno-Blot^®^ PVDF Membrane; Trans-Blot^®^ Turbo™ Transfer System, Bio-Rad Laboratories, Hercules, CA, USA). Membranes were blocked for 2 h in Tris-buffered saline (TBS) containing non-fat dry milk (5%) and Tween 20 (0.1%) (TBS-T) at room temperature (RT). Membranes were washed with TBS-T (3 times, 5 min/wash) and incubated overnight at 4 °C with any one of the primary antibodies (Supplementary Table S2). Membranes were washed with TBS-T (3 times, 5 min/wash) and incubated at RT for 4 h with suitable alkaline phosphatase (ALP)-conjugated secondary antibody (Supplementary Table S2). Membranes were washed with TBS-T (2 times, 5 min/wash). ALP activity was visualized using a commercial kit (Cat. #ES006; AP Detection Reagent Kit; Merck Life Sciences, Darmstadt, Germany). Images were captured, and band intensities were quantified using the ChemiDoc XRS+ System with Image Lab 2.0 software (Bio-Rad Laboratories, Hercules, CA, USA). Data were normalized to β-actin (for total proteins) or histone H3 (for histones). Full blot images are provided in the Supplementary Material.

### 4.12. Statistical Analysis

One-way/two-way analyses of variance (ANOVA) were performed for behavioural data analyses, and one-way ANOVA for molecular data using SigmaStat (version 11.0), to assess differences among experimental groups, followed by Bonferroni post hoc tests. Data are presented as mean ± standard error of the mean (SEM) and were graphed using GraphPad Prism (version 8.0). Significant differences are indicated as * *p* < 0.05, ** *p* < 0.01, and *** *p* < 0.001; not significant differences are denoted as NS.

## 5. Conclusions

The observed motor function deficits are accompanied by molecular alterations in the cortex, including elevated levels of inflammatory markers (C5a, IL-6), matrix metalloproteinases (MMP-2, MMP-3, MMP-9) and epigenetic regulators (DNMT3A, MeCP2, H3K4me3), as well as reduced levels of reelin and PSD-95. Importantly, the inhibition of MMP regulates the pathological changes, i.e., motor function and normalizing the molecular profile. This study highlights that the infection-induced neuroinflammatory signal during brain development regulates the methylation of *Reln* and associated synaptic plasticity in motor coordination.

## Data Availability

The original contributions presented in this study are included in the article and Supplementary Materials. Further inquiries can be directed to the corresponding authors.

## Supplementary Materials

The following supporting information can be downloaded at: https://www.mdpi.com/article/10.3390/ijms27093761/s1.
