# Supplementary material for: Maternal Infection Impairs Motor Coordination in an Experimental Meningitis Rat Model Through Altered MMP-2/3/9 Activity, H3K4 Trimethylation, and Reln Methylation
Source: Int J Mol Sci. 2026 Apr 23;27(9):3761. doi: 10.3390/ijms27093761 (PMC13164342; doi:10.3390/ijms27093761)
Supplement: Supplementary file 1 [file ijms-27-03761-s001.zip › ijms-4251090-supplementary.pdf]

Supplementary Information

Maternal infection impairs motor coordination in an experimental meningitis rat model through altered MMP-2/3/9 activity, H3K4 trimethylation, and *Reln* methylation

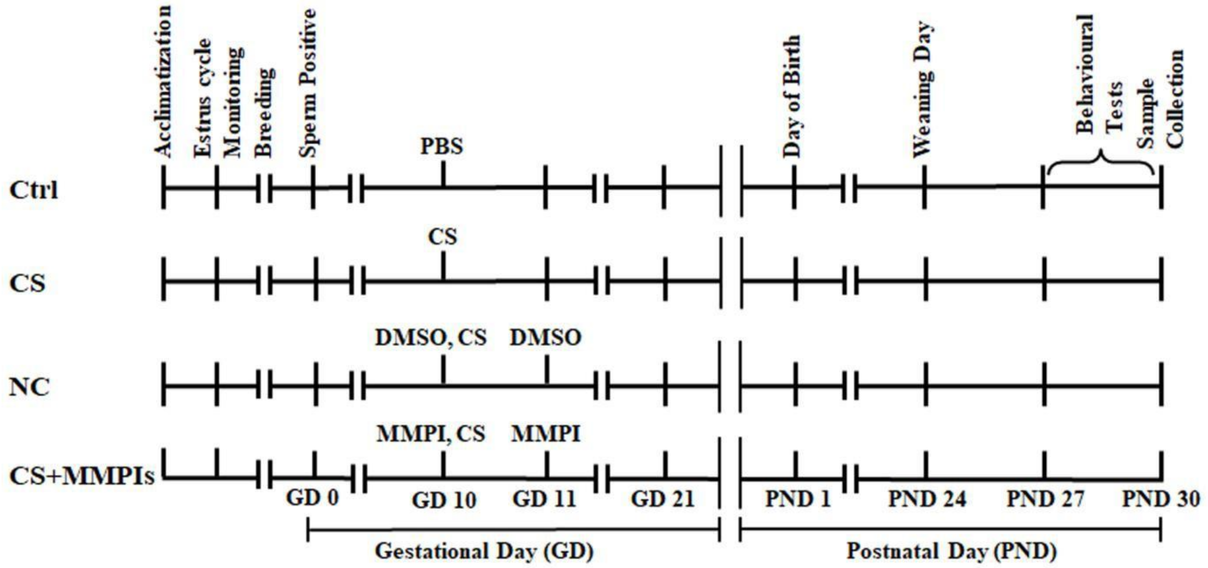

Supplementary Figure S1. Schematic representation of the timeline describing the experimental procedures for the Control group (Ctrl, no treatment), *Cronobacter sakazakii*-infected group (CS, 10^7 CFU *C. sakazakii*), Negative Control group (NC, 10^7 CFU *C. sakazakii* + 500µL DMSO on gestational day (GD)10 and 500µL DMSO on GD 11), and CS+MMPIs [10^7 CFU CS + MMPI (BB-94) on GD10 and another dosage of BB-94 on GD11). Experimental group rat pups were separated from their mother on post-natal day (PND) 24. Behavioural tests were conducted between PND 27 and PND 30. Samples were collected on PND 30.

Supplementary Table S1
Details of primers used in quantitative real-time PCR.

Reln unmethylated	Forward	5′-TGTTAAATTTTTGTAGTATTGGGGATGT-3′
	Reverse	5′-TCCTTAA AATAATCCAACAACACACC-3′
Reln methylated	Forward	5′-GGTGTTA AATTTTTGTAGTATTGGGGAC-3′
	Reverse	5′-TCCTTAAAATAATCCAACAACACGC-3′
Reln mRNA	Forward	5′-AAACTACAGCGGGTGGAACC-3′
	Reverse	5′-ATTTGAGGCATGACGGACCTATAT-3′
β-actin mRNA	Forward	5′-AGCAGATGTGGATCACAAGC-3′
	Reverse	5′-GCTCAGTAACAGTCCG CCTAGA-3′

Supplementary Table S2

Details of primary and secondary antibodies used in Western blot.

S. No.	Name of the Antibody	Cat. No.	Company	Dilution	Details
1.	Anti-β-actin mouse polyclonal antibody	sc-130656	Santa Cruz Biotechnology, Inc.	1:2000	Primary
2.	Anti-ADAMTS1 rabbit polyclonal antibody	PG-92403	Puregene	1:2000	Primary
3.	Anti-IL6 rabbit polyclonal antibody	PG-59003	Puregene	1:2000	Primary
4.	Anti-MMP2 mouse monoclonal antibody (1H1)	ABM40321	Abbkine Scientific Co., Ltd.	1:2000	Primary
5.	Anti-Cleaved MMP-3 (F100) rabbit polyclonal antibody	ABP50050	Abbkine Scientific Co., Ltd.	1:2000	Primary

6.	Anti-MMP9 rabbit polyclonal antibody	ABP53088	Abbkine Scientific Co., Ltd.	1:2000	Primary
7.	Anti-PSD95 rabbit polyclonal antibody	AF5283	Affinity Biosciences	1:2000	Primary
8.	Anti-RELN rabbit polyclonal antibody	ABP60127	Abbkine Scientific Co., Ltd.	1:2000	Primary
9.	Anti-DNMT3A rabbit polyclonal antibody	PG-88201	Puregene	1:2000	Primary
10.	Anti-MeCP2 rabbit monoclonal antibody	3456	Cell Signaling Technology, Inc.	1:2000	Primary
11.	Anti-Histone H3 rabbit monoclonal antibody	4499	Cell Signaling Technology, Inc.	1:2000	Primary
12.	Anti-H3K4me3 rabbit monoclonal antibody	9751	Cell Signaling Technology, Inc.	1:2000	Primary
13.	Goat Anti-rabbit IgG ALP	1100180011730	GeNei^{TM}	1:5000	Secondary
14.	Goat Anti-mouse IgG ALP	1100480011730	GeNei^{TM}	1:5000	Secondary

a

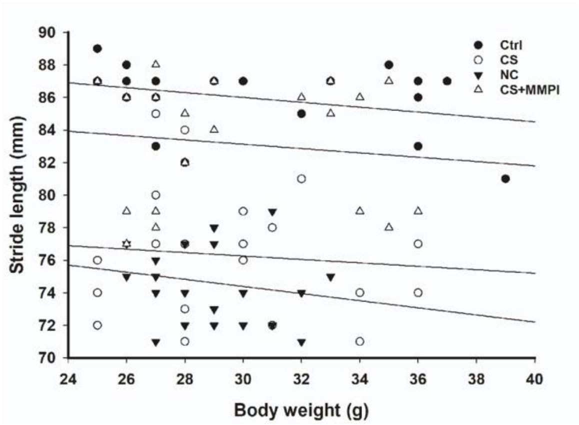

b

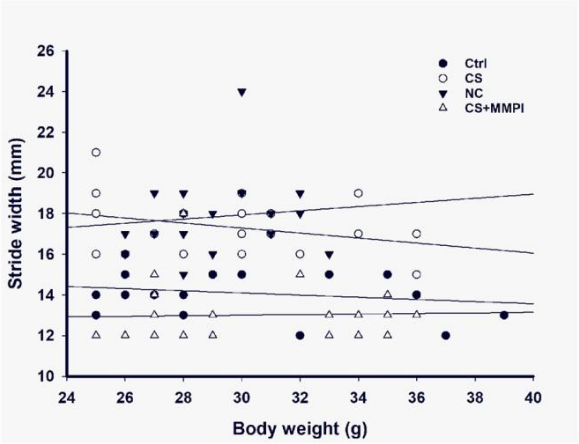

Supplementary Figure S2. Scatter plots showing the correlation between offspring's body weight and gait parameters: (a) stride length and (b) stride width. The regression line is shown for each group's gait parameter correlation pattern with body weight.

Supplementary Table S3

Detailed statistical data of one-way ANOVA used in Figure 1.

Figure	Behavioural parameter	Interaction between groups		Interaction within group	
		F-value	*P*-value	Pairwise comparison	P-value
Fig. 1a	Stride length (SL)	$F_{(3,87)}$=104.133	*P*<0.001	Ctrl vs. CS	*P*<0.001
				Ctrl vs. NC	*P*<0.001
				Ctrl vs. CS+MMPIs	*P*<0.001
				CS. vs. NC	*P*=1.000
				CS vs. CS+MMPIs	*P*<0.001
				NC vs. CS+MMPIs	*P*<0.001
Fig. 1b	Stride width (SW)	$F_{(3,87)}$=58.993	*P*<0.001	Ctrl vs. CS	*P*<0.001
				Ctrl vs. NC	*P*<0.001
				Ctrl vs. CS+MMPIs	*P*=0.090
				CS. vs. NC	*P*=1.000
				CS vs. CS+MMPIs	*P*<0.001
				NC vs. CS+MMPIs	*P*<0.001

Supplementary Table S4

Detailed statistical data of two-way ANOVA used in Figure 2.

Figure	Interaction with Experimental Groups	Interaction with FL/HL	Interaction with experimental groups and FL/HL	Comparison	*P*-value
Fig. 2a	$F_{(3,175)}$=517.335 *P*<0.001	$F_{(1,175)}$=1.105 *P*=0.295	$F_{(3,175)}$=0.719 *P*=0.542	CS vs. Ctrl	*P*<0.001
				CS vs. CS+MMPIs	*P*<0.001
				CS vs. NC	*P*<0.001
				NC vs. Ctrl	*P*=1.000
				NC vs. CS+MMPIs	*P*<0.001
				CS+MMPIs vs. Ctrl	*P*=1.000
Fig. 2b	$F_{(3,175)}$=680.272 *P*<0.001	$F_{(1,175)}$=5.750 *P*=0.018	$F_{(3,175)}$=3.040 *P*=0.031	CS vs. Ctrl	*P*<0.001
				CS vs. CS+MMPIs	*P*<0.001
				CS vs. NC	*P*<0.001
				NC vs. Ctrl	*P*=1.000
				NC vs. CS+MMPIs	*P*<0.001
				CS+MMPIs vs. Ctrl	*P*=1.000

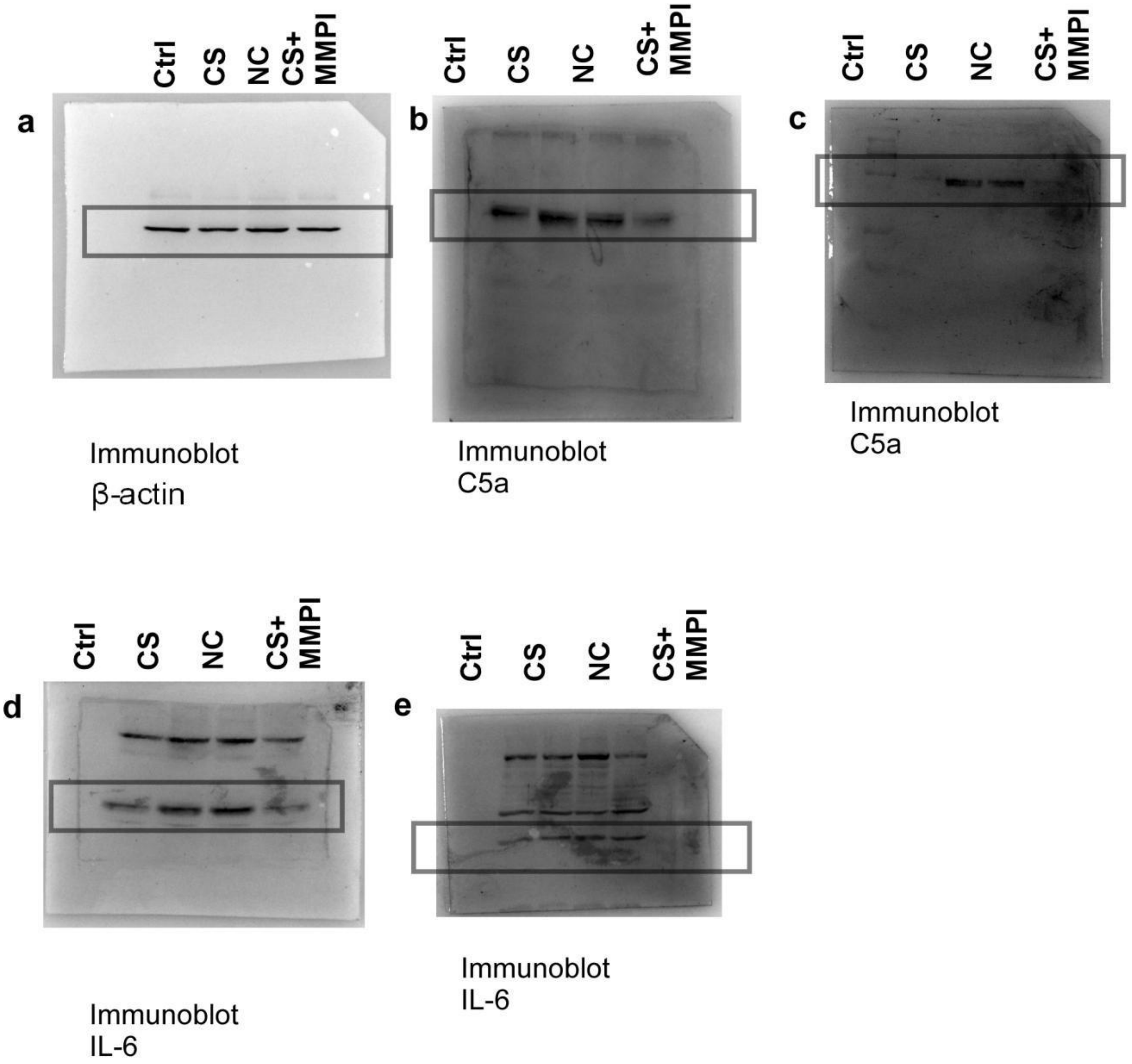

Supplementary Figure S3. Full immunoblot (uncropped) of (a) β-actin, (b) C5a; (c) IL-6 for Figure 3 in the manuscript. Gray rectangles are section of the images cropped from each blot that are shown in the manuscript image and each lane representing experimental groups Control (Ctrl); *C. sakazakii* infection (CS) [received a single intravaginal dose of 10^7 CFU on GD-10]; Negative Control (NC), which received 50 μL of 5% dimethyl sulfoxide (DMSO) intraperitoneally 1 h before infection on GD-10 and 24 h after infection on GD-11; *C. sakazakii* + Matrix Metalloproteinase Inhibitors (CS + MMPIs), which received Batimastat (50 mg/kg; Cat. #CAS130370-60-4; Calbiochem, Sigma-Aldrich) dissolved in 5% DMSO intraperitoneally 1 h before on GD-10 and 24 h after intravaginal *C. sakazakii* administration on GD-11.

Supplementary Table S5

Detailed statistical data of one-way ANOVA used in Figure 3.

Fig. 3b	C5a	$F_{(3,23)}$=87.212	*P* <0.001	Ctrl vs. CS	*P*<0.001
				Ctrl vs. NC	*P*<0.001
				Ctrl vs. CS+MMPIs	*P*<0.001
				CS. vs. NC	*P*=1.000
				CS vs. CS+MMPIs	*P*<0.001
				NC vs. CS+MMPIs	*P*<0.001
Fig. 3c	IL6	$F_{(3,23)}$=71.467	*P* <0.001	Ctrl vs. CS	*P*<0.001
				Ctrl vs. NC	*P*<0.001
				Ctrl vs. CS+MMPIs	*P*=0.504
				CS. vs. NC	*P*=0.351
				CS vs. CS+MMPIs	*P*<0.001
				NC vs. CS+MMPIs	*P*<0.001

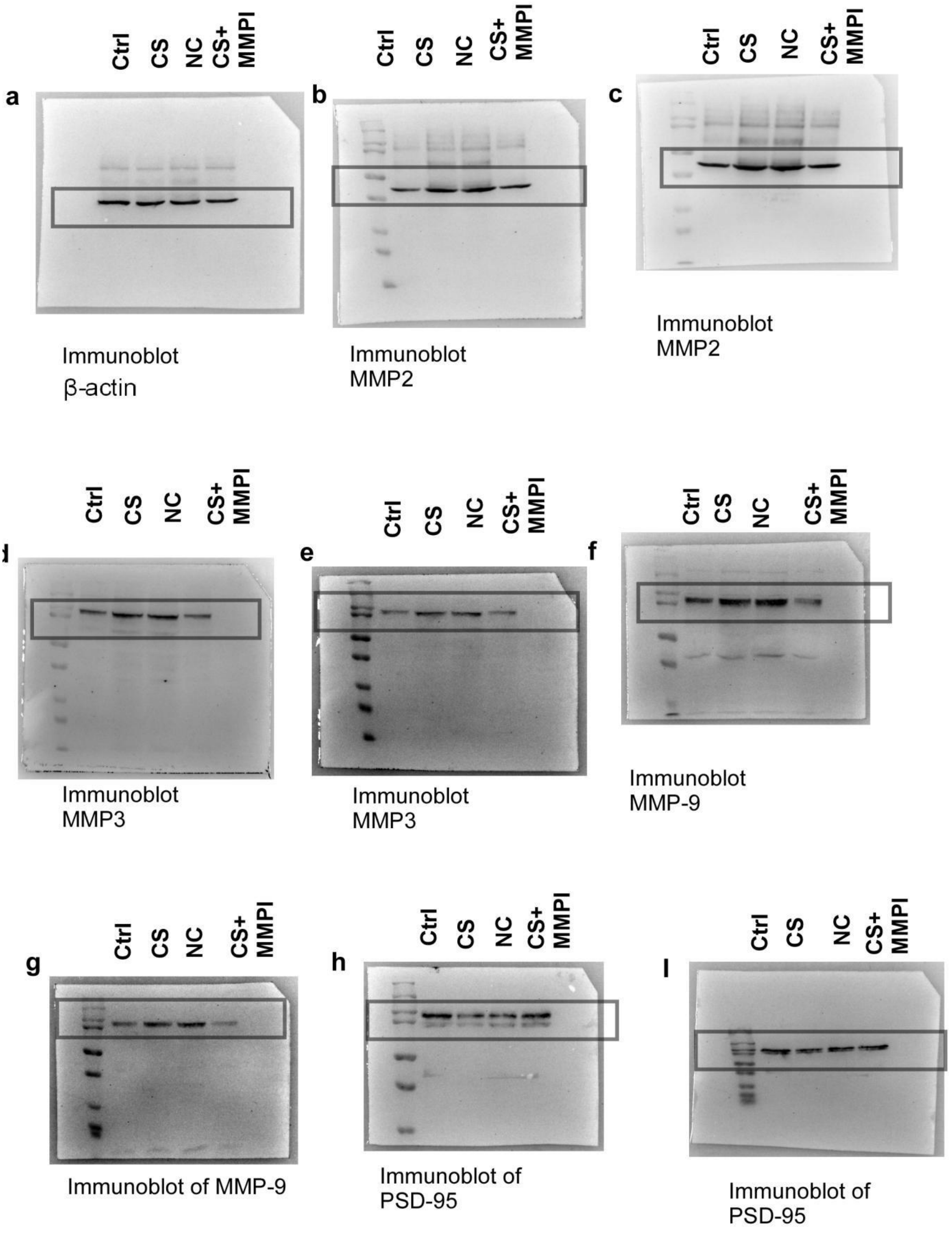
a
Ctrl
CS
NC
CS+ MMPI
Immunoblot
β-actin
b
Ctrl
CS
NC
CS+ MMPI
Immunoblot
MMP2
c
Ctrl
CS
NC
CS+ MMPI
Immunoblot
MMP2
d
Ctrl
CS
NC
CS+ MMPI
Immunoblot
MMP3
e
Ctrl
CS
NC
CS+ MMPI
Immunoblot
MMP3
f
Ctrl
CS
NC
CS+ MMPI
Immunoblot
MMP-9
g
Ctrl
CS
NC
CS+ MMPI
Immunoblot of MMP-9
h
Ctrl
CS
NC
CS+ MMPI
Immunoblot of
PSD-95
I
Ctrl
CS
NC
CS+ MMPI
Immunoblot of
PSD-95

Supplementary Figure S4. Full immunoblot (uncropped) of (a) β-actin (b,c) MMP-2; (d,e) MMP-3; (f,g) MMP-9; (h,i) PSD-95 used for Figure 4 in the manuscript. Gray rectangles are the section of the images cropped from each blot that are shown in the manuscript image and each lane representing experimental groups Control (Ctrl); *C. sakazakii* infection (CS) [received a single intravaginal dose of 10^7 CFU on GD-10]; Negative Control (NC), which received 50 μL of 5% dimethyl sulfoxide (DMSO) intraperitoneally 1 h before infection on GD-10 and 24 h after infection on GD-11; *C. sakazakii* + Matrix Metalloproteinase Inhibitors (CS + MMPIs), which received Batimastat (50 mg/kg; Cat. #CAS130370-60-4; Calbiochem, Sigma-Aldrich) dissolved in 5% DMSO intraperitoneally 1 h before on GD-10 and 24 h after intravaginal *C. sakazakii* administration on GD-11.

Supplementary Table S6

Detailed statistical data of one-way ANOVA used in Figure 4.

Fig. 4b	MMP2	$F_{(3,23)}$=34.905	*P* <0.001	Ctrl vs. CS	*P*<0.001
				Ctrl vs. NC	*P*<0.001
				Ctrl vs. CS+MMPIs	*P*=0.936
				CS. vs. NC	*P*=1.000
				CS vs. CS+MMPIs	*P*<0.001
				NC vs. CS+MMPIs	*P*<0.001
Fig. 4c	MMP3	$F_{(3,23)}$=332.247	*P* <0.001	Ctrl vs. CS	*P*<0.001
				Ctrl vs. NC	*P*<0.001
				Ctrl vs. CS+MMPIs	*P*=0.060
				CS. vs. NC	*P*=0.263
				CS vs. CS+MMPIs	*P*<0.001
				NC vs. CS+MMPIs	*P*<0.001
Fig. 4d	MMP9	$F_{(3,23)}$=104.441	*P* <0.001	Ctrl vs. CS	*P*<0.001
				Ctrl vs. NC	*P*<0.001
				Ctrl vs. CS+MMPIs	*P*=1.000
				CS. vs. NC	*P*=1.000
				CS vs. CS+MMPIs	*P*<0.001
				NC vs. CS+MMPIs	*P*<0.001
Fig. 4e	PSD95	$F_{(3,23)}$=100.302	*P* <0.001	Ctrl vs. CS	*P*<0.001
				Ctrl vs. NC	*P*<0.001
				Ctrl vs. CS+MMPIs	*P*=1.000
				CS. vs. NC	*P*=0.400
				CS vs. CS+MMPIs	*P*<0.001
				NC vs. CS+MMPIs	*P*<0.001

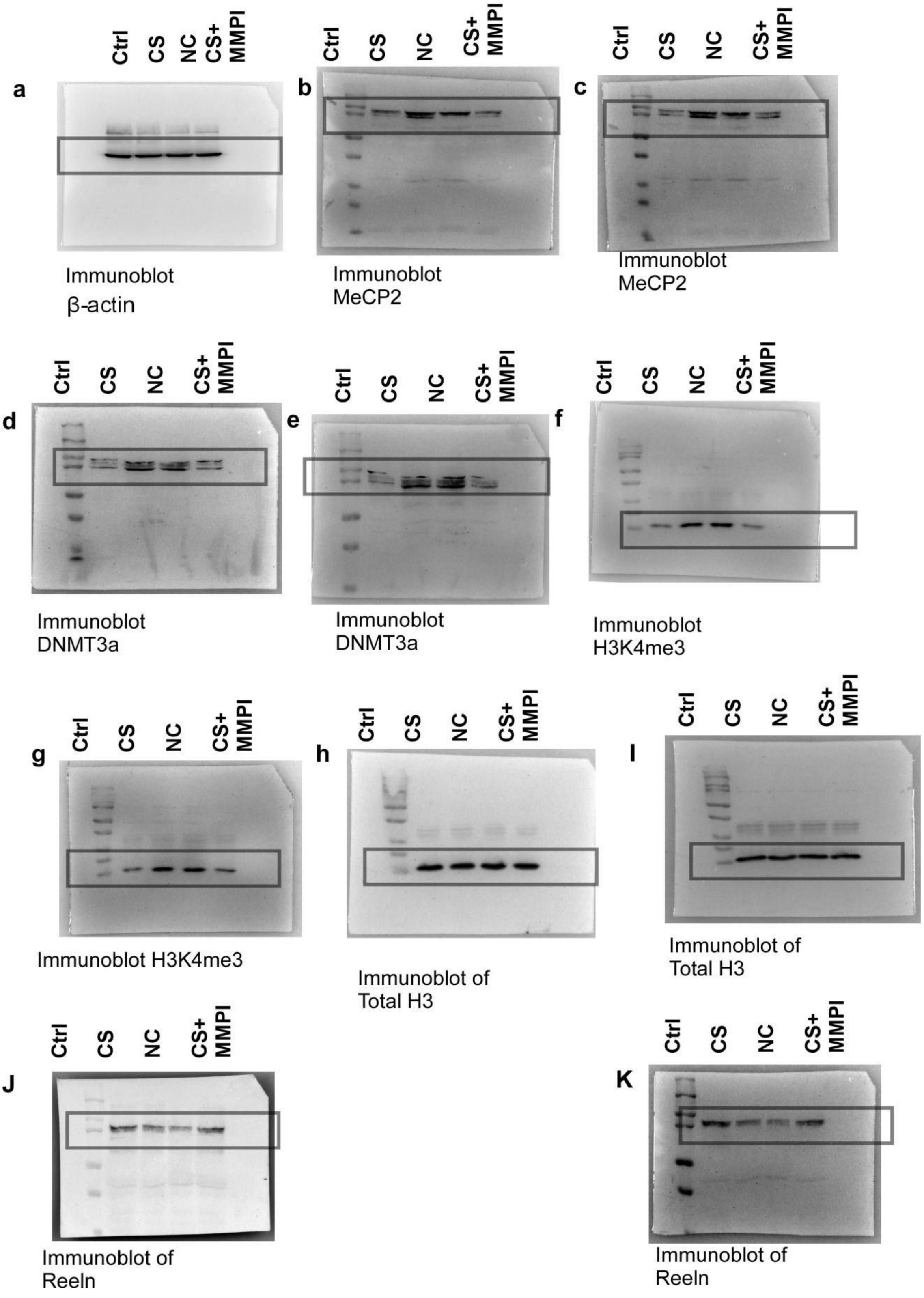
a
b
c
d
e
f
g
h
I
J
K
Ctrl
CS
NC
CS+ MMPI
Immunoblot β-actin
Immunoblot MeCP2
Immunoblot MeCP2
Immunoblot DNMT3a
Immunoblot DNMT3a
Immunoblot H3K4me3
Immunoblot H3K4me3
Immunoblot of Total H3
Immunoblot of Total H3
Immunoblot of Reeln
Immunoblot of Reeln

Supplementary Figure S5. Full immunoblot (uncropped) of (a) β-actin (b,c) MeCP2; (d,e) DNMT3a; (f,g) H3K4me3; (h,i) total H3; (j,k) Reln used for Figure 5 in the manuscript. Gray rectangles are the section of the images cropped from each blot that are shown in the manuscript image and each lane representing experimental groups Control (Ctrl); *C. sakazakii* infection (CS) [received a single intravaginal dose of 10^7 CFU on GD-10]; Negative Control (NC), which received 50 μL of 5% dimethyl sulfoxide (DMSO) intraperitoneally 1 h before infection on GD-10 and 24 h after infection on GD-11; *C. sakazakii* + Matrix Metalloproteinase Inhibitors (CS + MMPIs), which received Batimastat (50 mg/kg; Cat. #CAS130370-60-4; Calbiochem, Sigma-Aldrich) dissolved in 5% DMSO intraperitoneally 1 h before on GD-10 and 24 h after intravaginal *C. sakazakii* administration on GD-11.

Supplementary Table S7

Detailed statistical data of one-way ANOVA used in Figure 5.

Fig. 5b	DNMT3A	$F_{(3,23)}$=165.298	*P* <0.001	Ctrl vs. CS	*P*<0.001
				Ctrl vs. NC	*P*<0.001
				Ctrl vs. CS+MMPIs	*P*=2.969
				CS. vs. NC	*P*=1.000
				CS vs. CS+MMPIs	*P*<0.001
				NC vs. CS+MMPIs	*P*<0.001
Fig. 5c	MeCP2	$F_{(3,23)}$=88.050	*P* <0.001	Ctrl vs. CS	*P*<0.001
				Ctrl vs. NC	*P*<0.001
				Ctrl vs. CS+MMPIs	*P*<0.001
				CS. vs. NC	*P*=0.192
				CS vs. CS+MMPIs	*P*<0.001
				NC vs. CS+MMPIs	*P*<0.001
Fig. 5d	H3K4me3	$F_{(3,23)}$=247.679	*P* <0.001	Ctrl vs. CS	*P*<0.001
				Ctrl vs. NC	*P*<0.001
				Ctrl vs. CS+MMPIs	*P*=1.000
				CS. vs. NC	*P*=0.625
				CS vs. CS+MMPIs	*P*<0.001
				NC vs. CS+MMPIs	*P*<0.001
Fig. 5f	*Reln* mRNA	$F_{(3,13)}$=8.361	*P* =0.008	Ctrl vs. CS	*P*=0.015
				Ctrl vs. NC	*P*=0.011
				Ctrl vs. CS+MMPIs	*P*=0.270
				CS. vs. NC	*P*=0.550
				CS vs. CS+MMPIs	*P*=0.037

				NC vs. CS+MMPIs	*P*=0.034
Fig. 5e	Met/unmet level of *Reln*	$F_{(3,23)}$=30.762	*P* <0.001	Ctrl vs. CS	*P*<0.001
				Ctrl vs. NC	*P*<0.001
				Ctrl vs. CS+MMPIs	*P*=1.000
				CS. vs. NC	*P*=0.313
				CS vs. CS+MMPIs	*P*<0.001
				NC vs. CS+MMPIs	*P*<0.001
Fig. 5g	Reelin	$F_{(3,23)}$=23.234	*P* <0.001	Ctrl vs. CS	*P*<0.001
				Ctrl vs. NC	*P*<0.001
				Ctrl vs. CS+MMPIs	*P*=1.000
				CS. vs. NC	*P*=1.000
				CS vs. CS+MMPIs	*P*<0.001
				NC vs. CS+MMPIs	*P*<0.001
